# Genetic Variants of *MICB* and *PLCE1* and Associations with Non-Severe Dengue

**DOI:** 10.1371/journal.pone.0059067

**Published:** 2013-03-11

**Authors:** James Whitehorn, Tran Nguyen Bich Chau, Nguyen Minh Nguyet, Duong Thi Hue Kien, Nguyen Than Ha Quyen, Dinh The Trung, Junxiong Pang, Bridget Wills, Nguyen Van Vinh Chau, Jeremy Farrar, Martin L. Hibberd, Chiea Chuen Khor, Cameron P. Simmons

**Affiliations:** 1 Department of Clinical Research, London School of Hygiene and Tropical Medicine, London, United Kingdom; 2 Oxford University Clinical Research Unit, Hospital for Tropical Diseases, Ho Chi Minh City, Viet Nam; 3 Genome Institute of Singapore, Singapore, Singapore; 4 School of Public Health, National University of Singapore, Singapore, Singapore; 5 Centre for Tropical Medicine, University of Oxford, Oxford, United Kingdom; 6 Hospital for Tropical Diseases, Ho Chi Minh City, Viet Nam; 7 Department of Paediatrics, National University of Singapore, Singapore, Singapore; 8 Department of Ophthalmology, National University of Singapore, Singapore, Singapore; National Cancer Institute, National Institutes of Health, United States Of America

## Abstract

**Background:**

A recent genome-wide association study (GWAS) identified susceptibility loci for dengue shock syndrome (DSS) at *MICB* rs3132468 and *PLCE1* rs3740360. The aim of this study was to define the extent to which *MICB* (rs3132468) and *PLCE1* (rs3740360) were associated with less severe clinical phenotypes of pediatric and adult dengue.

**Methods:**

3961 laboratory-confirmed dengue cases and 5968 controls were genotyped at *MICB* rs3132468 and *PLCE1* rs3740360. Per-allele odds ratios (OR) with 95% confidence intervals (CI) were calculated for each patient cohort. Pooled analyses were performed for adults and paediatrics respectively using a fixed effects model.

**Results:**

Pooled analysis of the paediatric and adult cohorts indicated a significant association between *MICB* rs3132468 and dengue cases without shock (OR  =  1.15; 95%CI: 1.07 – 1.24; *P*  =  0.0012). Similarly, pooled analysis of pediatric and adult cohorts indicated a significant association between dengue cases without shock and *PLCE1* rs3740360 (OR  =  0.92; 95%CI: 0.85 – 0.99; *P*  =  0.018). We also note significant association between both SNPs (OR  =  1.48; P  =  0.0075 for *MICB* rs3132468 and OR  =  0.75, *P*  =  0.041 for *PLCE1* rs3740360) and dengue in infants.

**Discussion:**

This study confirms that the *MICB* rs3132468 and *PLCE1* rs3740360 risk genotypes are not only associated with DSS, but are also associated with less severe clinical phenotypes of dengue, as well as with dengue in infants. These findings have implications for our understanding of dengue pathogenesis.

## Introduction

Dengue is the most important arboviral disease of humans.[1] Dengue viruses (DENV) cause a spectrum of clinical manifestations ranging from asymptomatic infection through to life-threatening shock and haemorrhage.[1], [2] The clinical outcome of an individual infection is influenced by a variety of virus and host-related factors. The host factors that influence the clinical course of an individual infection include flavivirus infection history, host genotype, sex, age, and the presence of underlying medical conditions.[3]–[5] The first GWAS in dengue identified susceptibility loci for dengue shock syndrome (DSS) at MHC class I polypeptide-related sequence B (*MICB*) (C/T, rs3132468) on chromosome 6 and phospholipase C, epsilon 1 (*PLCE1*) (C/A, rs3740360) on chromosome 10.[6] The *MICB* gene encodes an activating ligand of natural killer (NK) cells (and possibly CD8+ T cells). We have previously speculated that mutations in *MICB* might result in impaired induction of anti-viral effector functions in NK cells with the consequence being a greater DENV-infected cell mass in vivo [6], a recognised risk factor for severe dengue.[7]

The identification of variants of *PLCE1* as being associated with severe dengue is intriguing.[6] Rare mutations of high penetrance within *PLCE1* are associated with nephrotic syndrome, a condition characterised by oedema secondary to proteinuria and reduced vascular oncotic pressure.[8] Since plasma leak, proteinuria and hypovolemia are also characteristic features of severe dengue, it’s plausible that nephrotic syndrome and severe dengue share some common underlying pathophysiological processes. Furthermore, there are data implicating *PLCE1* in the homeostatic regulation of blood pressure.[9] These findings have the potential to help us define more clearly the functional basis of *PLCE1* variants in severe dengue.

The SNP associations identified at *MICB* (rs3132468) and *PLCE1* (rs3740360) by the GWAS study were in the context of pediatric patients with DSS, leaving unanswered the question whether they are also associated with less severe clinical phenotypes of dengue. To this end, the aim of this study was to define the extent to which these alleles were associated with milder clinical phenotypes of pediatric and adult dengue. We analyzed a total of 3961 laboratory-confirmed dengue cases, independent from the initial GWAS study, and 5968 cord blood controls.[6]

## Materials and Methods

### Ethics statement

All participants gave written informed consent to participate in the prospective studies summarised in **Table 1** (details of the inclusion and exclusion criteria are available in **Supplementary** **Table 1**). Parents or guardians provided written informed consent on behalf of the children involved in the studies. The protocols for these studies were approved by the Institutional Review Boards of each study site (Hospital for Tropical Diseases HCMC, Children’s Hospital 1 and 2 HCMC, Hung Vuong Hospital HCMC, Dong Thap Hospital, Sa Dec Hospital and Tien Giang Hospital) and by the Oxford University Tropical Research Ethics Committee. Each ethical committee approved of the consent procedure detailed above.

**Table 1:** Summary of the cohort studies used in the analysis.

Group	Number (% male)	Median age (range 5^th^ – 95^th^)
Control	1068 (52)	At birth
Infants	165 (55)	7 months (3 – 12 months)
DC	59 (52)	6 (3 – 11)
DT	88 (57)	7 (4 – 11)
DZ	8 (50)	8 (7 – 11)
FB	9 (55)	8 (7 – 15)
Children/Young adults	2759 (56)	12 years (6 – 16 years)
FG	576 (56)	11 (4 – 22)
06DX	220 (71)	13 (11 – 14)
DR	582 (56)	11 (6 – 15)
MD	1381 (60)	12 (7 – 14)
Adults	741 (46)	22 (15 – 35)
09DX	159 (41)	23 (19 – 27)
D001	54 (43)	20 (14 – 25)
FL	528 (48)	22 (15 – 35)

### Enrolment and diagnosis

Blood samples for genotyping were collected in one of several prospective studies of dengue in Vietnamese patients detailed in **Table 1**. Dengue cases were laboratory-confirmed by one of three methods: IgM-seroconversion by ELISA assay on paired samples, detection of DENV RNA by RT-PCR, or detection of non-structural protein 1 (NS1) by ELISA (BioRad Platelia). The control samples used in this study were from blood samples collected from the umbilical cord of newborn infants enrolled into the birth cohort study detailed in Table 1. Within each cohort, dengue cases were classified in a binary fashion as being “DSS” or “not-DSS”.[6] Consistent with the original GWAS study, DSS cases were defined as laboratory-confirmed dengue cases with cardiovascular decompensation secondary to plasma leakage and requiring fluid resuscitation.[2]

### Genotyping

DNA extractions were performed using a MagNA Pure 96 DNA and Viral NA Small Volume Kit (Roche, Germany) according to the manufacturer’s instructions. Candidate SNPs were genotyped using a TaqMan^®^ genotyping assay to amplify and detect the specific alleles in the DNA samples as per manufacturer instructions.

### Statistical analysis

The data were analysed using PLINK version 1.07 and the R statistical software package version 2.12.0 (2010 The R Foundation for Statistical Computing). For each cohort study per-allele odds ratios with 95% confidence intervals were calculated to assess the relationship between SNP genotypes (rs3132468 at *MICB* and rs3740360 at *PLCE1*) and susceptibility to dengue. For the analysis we considered non-DSS cases in children separately from adults and considered infants with dengue as a distinct group. Infants were defined as less than 1 year in age, children were defined by ages between 1 and 15 years, and adults were defined by being 16 years and older. Other variables known to be associated with disease severity were not included in this analysis, consistent with the GWAS primary analysis for DSS as well as other infectious diseases.[6], [10]–[12] Pooled odds ratios across the different sample collections were calculated using the inverse-variance, fixed effects model, as previously described.[13] This model used the weighted average of each study’s odds ratio. The weights used were the inverse of the variance of the study’s estimated odds ratio, ensuring that larger studies command greater weight. Forest plots demonstrating these results were created in R.

## Results

### Case and control cohorts

**Figure 1:**
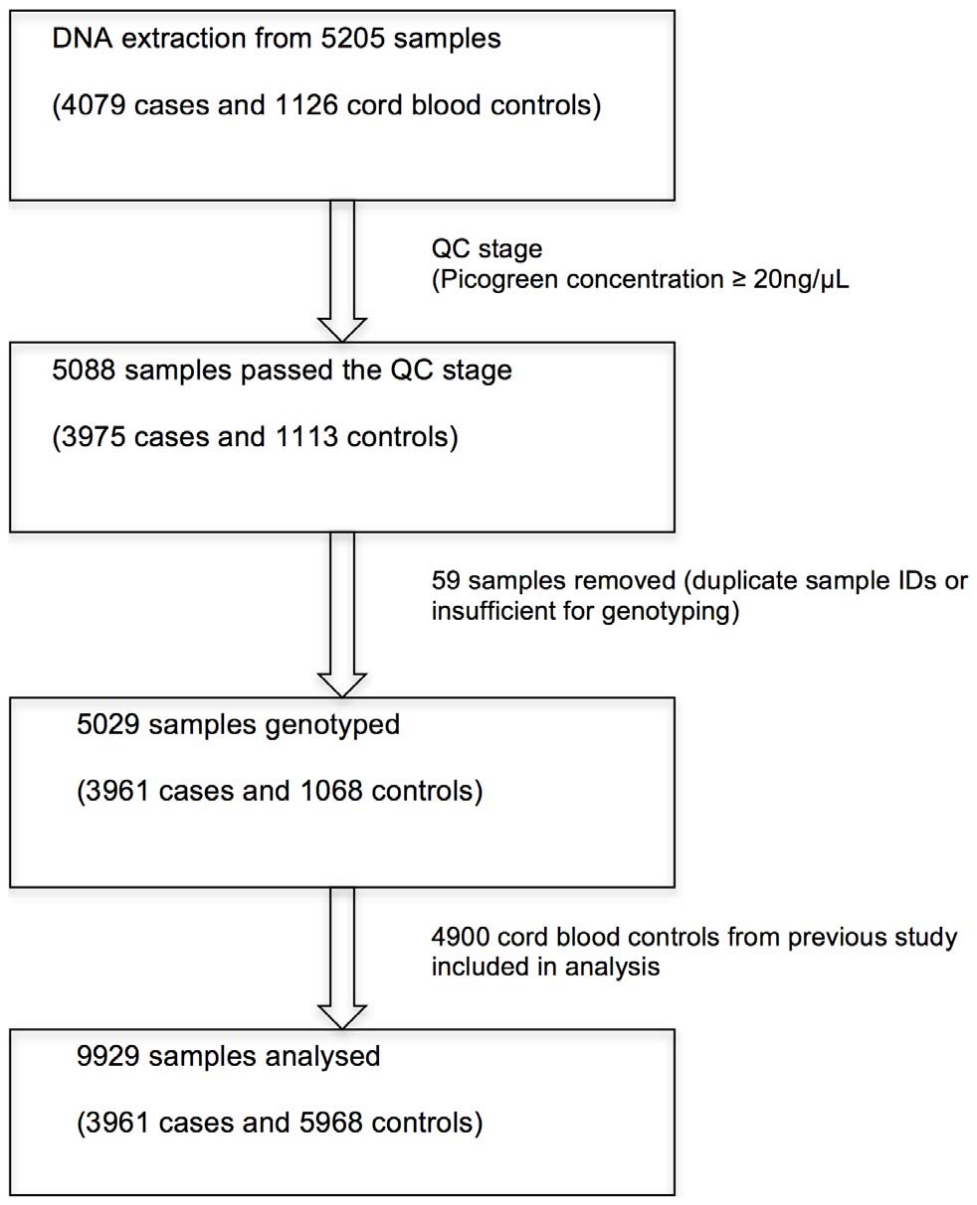
Genotyping and sample quality control process flowchart.

The patient cohorts that provided the laboratory-confirmed dengue cases that were genotyped in this study are summarised in **Table 1**. Across the 7 cohorts (4 pediatric and 3 adult) there were 297 DSS cases (161 pediatric and 136 adult), 3500 non-DSS dengue cases (2759 pediatric and 741 adult cases), and 164 cases of infants with dengue that were successfully genotyped at *MICB* rs3132468 and *PLCE1* rs3740360. Umbilical cord blood DNA samples (n = 1068) were similarly genotyped at *MICB* rs3132468 and *PLCE1* rs3740360 and served as population controls. Data from this was complemented with genotype data from 4900 controls studied previously.[6] In *a priori* analyses we considered non-DSS cases in children separately from adults. Infants with dengue (n = 164) were also treated as a distinct cohort because of the unique context of dengue in this age group. The sample quality control process is illustrated in **Figure 1**.

### Association between MICB rs3132468 and dengue in children and adults

**Figure 2:**
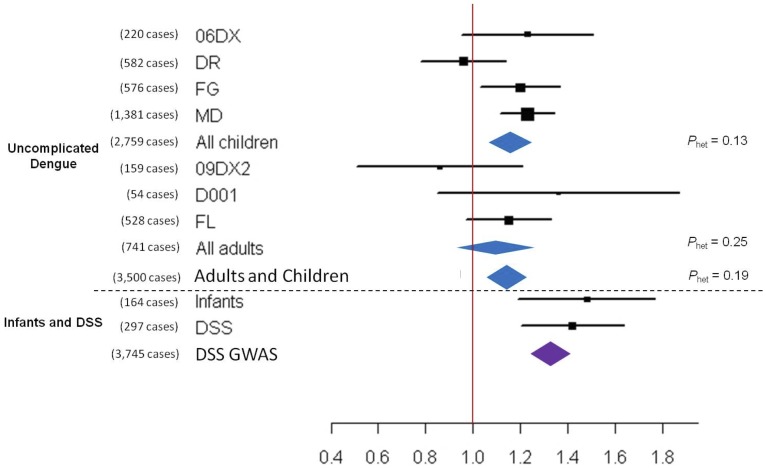
Forest plot illustrating the association between *MICB* rs3132468 and susceptibility to dengue. 06DX, DR, FG and MD were cohort studies of children, and 09DX, D001 and FL were cohort studies of adults. The oblongs represent point estimates (referring to the per-allele odds ratio, expressed on the horizontal axis), with the height of the oblongs inversely proportional to the standard error of the point estimates. Horizontal lines indicate the 95% confidence interval for each point estimate. Meta-analysis of children, adults, as well as children and adults with uncomplicated dengue are reflected by blue diamonds. Data from our previously reported GWAS on DSS is reflected by the purple diamond.[6] The width of the diamonds indicates their 95% confidence intervals. Each meta-analysis is accompanied by a test of heterogeneity between the sample collections summarized by it (expressed as *P*_het_).

Pooled analysis of genotype data from the pediatric patient cohorts revealed that non-DSS dengue cases were significantly more likely to carry the *MICB* risk allele rs3132468 than controls (per-allele odds ratio (OR)  =  1.16; 95%CI: 1.07 – 1.25). Pooled analysis of cohorts of adult non-DSS dengue cases revealed a similar pattern of effect with the *MICB* risk allele, but this did not reach statistical significance (OR  =  1.10, *P*  =  0.11; **Figure 2**, and **Table 2**). However, a pooled analysis of all pediatric and adult patient cohorts indicated a significant association (OR  =  1.15, *P*  =  0.0014; **Figure 2**, and **Table 2**) compared to controls, with no heterogeneity between children and adults (*P*_het_  =  0.19). In the relatively small number of adult and pediatric DSS cases (N  =  297), we were able to confirm the association and effect size reported in our previous GWAS (OR  =  1.42, *P*  =  0.0014; **Figure 2** and **Table 2** **)**.

**Table 2:** Per-collection analysis for *MICB* rs3132468.

Children							
**Collection**	**Number**	**MAF cases**	**MAF controls**	**OR**	**95% CI**	**Weight**	***P***
06DX	220	0.160	0.134	1.23	0.95 – 1.51	49.45393	0.14
DR	582	0.129	0.134	0.96	0.78 – 1.14	119.4422	0.66
FG	576	0.156	0.134	1.20	1.03 – 1.37	134.8943	0.035
MD	1381	0.159	0.134	1.23	1.12 – 1.35	289.2313	0.00047
All children	2759	0.152	0.134	1.16	1.07 – 1.25	-	0.0011
Adults							
**Collection**	**Number**	**MAF cases**	**MAF controls**	**OR**	**95% CI**	**Weight**	***P***
09DX2	159	0.118	0.134	0.86	0.51 – 1.21	31.03638	0.42
D001	54	0.173	0.134	1.36	0.85 – 1.87	14.72486	0.24
FL	528	0.151	0.134	1.15	0.97 – 1.33	120.2293	0.12
All adults	741	0.145	0.134	1.10	0.94 – 1.26	–	0.22
All mild cases (Adults and children)	3500	0.151	0.134	1.15	1.07 – 1.24	–	0.0014
**Collection**		**MAF cases**	**MAF controls**	**OR**	**95% CI**	**Weight**	***P***
All infants	164	0.186	0.134	1.48	1.19 – 1.77	–	0.0075
**Collection**		**MAF cases**	**MAF controls**	**OR**	**95% CI**	**Weight**	***P***
All DSS	297	0.180	0.134	1.42	1.20 – 1.64	–	0.0014

Number: Number of cases

MAF cases: minor allele frequency in dengue cases

MAF controls: minor allele frequency in controls

OR: per-allele odds ratio.

SE: Standard error of the OR

Weight: study-specific weight when meta-analyzed, not calculated for infants and DSS collections, which were not meta-analyzed.

*P*: *P*-value for association with disease (unadjusted).

### Association between PLCE1 rs3740360 and dengue in children and adults

**Figure 3:**
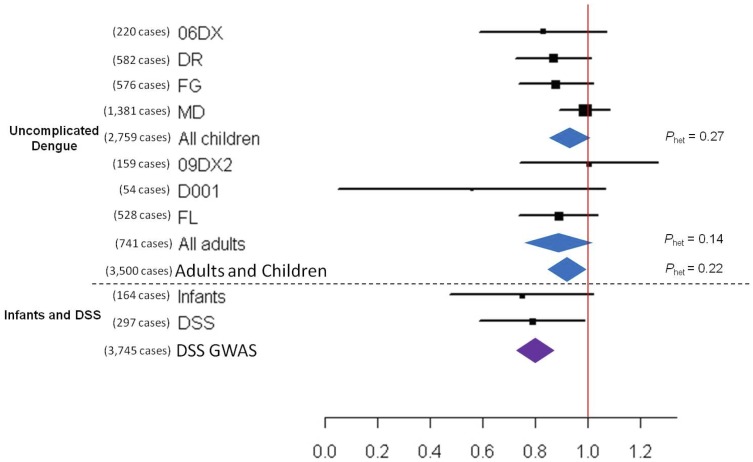
Forest plot illustrating the association between *PLCE1* rs3740360 and susceptibility to dengue. 06DX, DR, FG and MD were cohort studies of children. and 09DX, D001 and FL were cohort studies of adults. The oblongs represent point estimates (referring to the per-allele odds ratio, expressed on the horizontal axis), with the height of the oblongs inversely proportional to the standard error of the point estimates. Horizontal lines indicate the 95% confidence interval for each point estimate. Meta-analysis of children, adults, as well as children and adults with uncomplicated dengue are reflected by blue diamonds. Data from our previously reported GWAS on DSS is reflected by the purple diamond.[6] The width of the diamonds indicates their 95% confidence intervals. Each meta-analysis is accompanied by a test of heterogeneity between the sample collections summarized by it (expressed as *P*_het_).

We observed significant association with non-DSS cases upon pooled analysis of all adults and children (OR  =  0.92, *P*  =  0.018; **Figure 3** and **Table 3**). Amongst DSS cases, the association at *PLCE1* revealed by the previous GWAS was confirmed (OR  =  0.77: P  =  0.0094).

**Table 3:** Per-collection analysis for *PLCE1* rs3740360.

Children							
**Collection**	**Number**	**MAF cases**	**MAF controls**	**OR**	**95% CI**	**Weight**	***P***
06DX	220	0.235	0.271	0.83	0.59 – 1.07	66.09822	0.12
DR	582	0.244	0.271	0.87	0.73 – 1.01	190.7756	0.046
FG	576	0.248	0.271	0.88	0.74 – 1.02	189.726	0.089
MD	1381	0.270	0.271	0.99	0.90 – 1.08	426.8834	0.90
All children	2759	0.257	0.271	0.93	0.86 – 1.00		0.054
Adults							
**Collection**	**Number**	**MAF cases**	**MAF controls**	**OR**	**95% CI**	**Weight**	***P***
09DX2	159	0.272	0.271	1.01	0.75 – 1.27	56.10964	0.97
D001	54	0.173	0.271	0.56	0.05 – 1.07	14.7929	0.025
FL	528	0.249	0.271	0.89	0.74 – 1.04	171.3221	0.13
All adults	741	0.248	0.271	0.89	0.76 – 1.02		0.065
All mild cases (Adults and children)	3500	0.255	0.271	0.92	0.85 – 0.99		0.018
**Collection**		**MAF cases**	**MAF controls**	**OR**	**95% CI**	**Weight**	***P***
All infants	164	0.219	0.271	0.75	0.48 – 1.02		0.041
**Collection**		**MAF cases**	**MAF controls**	**OR**	**95% CI**	**Weight**	***P***
All DSS	297	0.223	0.271	0.77	0.59 – 0.99		0.0094

Number: Number of cases

MAF cases: minor allele frequency in dengue cases

MAF controls: minor allele frequency in controls

OR: per-allele odds ratio.

SE: Standard error of the OR

Weight: study-specific weight when meta-analyzed, not calculated for infants and DSS collections, which were not meta-analyzed.

*P*: *P*-value for association with disease (unadjusted).

### Association between MICB rs3132468 and dengue in infants

Since each of the infant cohorts was relatively small in their own right, a meta-analysis was performed. Consistent with the findings in older children, this pooled analysis revealed a significant association between dengue in infants and *MICB* rs3132468 (OR  =  1.48; *P*  =  0.0075), as well as *PLCE1* rs3740360. (OR  =  0.75, P  =  0.041) (**Figures 2** and **3** **;** **Tables 2** and **3**). Although the infant cohorts included 16 cases of DSS, removal of these samples did not affect the associations demonstrated.

## Discussion

*MICB* rs3132468 and *PLCE1* rs3740360 genotypes are associated with DSS in Vietnamese children.[6] Here, we have extended this finding by follow-up genotyping in a large number of Vietnamese adult and paediatric dengue cases, together with a new cohort of population control samples. The data revealed significant association between *MICB* rs3132468 and *PLCE1* rs3740360 and clinical phenotypes of dengue less severe than DSS, albeit with smaller effect sizes than observed between these alleles and DSS.[6] In addition, we observed association between both SNP genotypes and infants with dengue at effect sizes comparable to that seen with DSS. Finally, amongst children and adults with DSS in these cohorts, we were able to confirm association of *MICB* rs3132468 and *PLCE1* rs3740360 that was first observed in the previous GWAS.[6] Collectively, these findings provide further validation of the importance of *MICB* rs3132468 and *PLCE1* rs3740360 risk genotypes to shaping the clinical phenotype of dengue and raises intriguing questions about their roles in disease pathogenesis.

The association of the *MICB* rs3132468 genotype with clinical phenotypes of dengue less severe than DSS indicates a role for this variant in susceptibility to overall clinically apparent dengue and not just severe disease. Given the role of *MICB* in activation of NK and CD8^+^ cells these findings support a central role for these cell types in shaping the outcome of DENV infection. For example, it is plausible that the *MICB* rs3132468 genotype is associated with an impaired NK cell response, potentially resulting in a higher *in vivo* virus titre and an increased risk of developing both symptomatic and severe dengue. Furthermore, inefficient induction of regulatory NK cells might result in dysregulated T cell responses that may also shape the clinical phenotype.[14]

The effect size between the *PLCE1* rs3740360 risk genotype and milder clinical phenotypes of dengue was less pronounced than that observed for DSS cases.[6] Whilst a degree of endothelial leak is likely to occur in most clinically evident DENV infections, patients with DSS experience the most severe vascular permeability.[15], [16] Our current data, together with the recently demonstrated association between *PLCE1* rs3740360 and DSS, may indicate a central role for *PLCE1* in the context of vascular leakage.[6] Further genetic fine mapping studies will be required to pinpoint functional mutations that could mechanistically explain the association between *PLCE1* and DSS. In doing so, we expect to contribute to the wider understanding of the role of *PLCE1* in health and disease, particularly in light of its association with nephrotic syndrome, regulation of blood pressure and esophageal cancer.[8], [9], [17] In light of the observed association with *PLCE1* and nephrotic syndrome it is interesting that the degree of proteinuria has been proposed as a potential predictor in determining which dengue patients are at risk of developing more severe disease.[18]

Infants with dengue were analysed independently of other patient populations. Primary infection in the context of waning maternal antibody levels, immunological immaturity and vulnerable physiology make infants with dengue a distinct group.[19] Our data shows association between *MICB* rs3132468 and dengue in infants, with effect sizes in keeping with that observed in DSS patients.[6] We speculate that this reflects a prominent role for innate immunity and particularly NK cells in controlling early viral infection in infants; impaired control of viral replication could be a risk factor for clinically apparent dengue in this age group. The effect size observed at *PLCE1* rs3740360 was also similar to that observed in DSS patients.[6] It has been noted that hospitalised infants with dengue represent a group with the highest risk of death, and it is thought that this is partly related to an intrinsically more permeable vascular endothelium in this age group.[4] In infants with dengue, carriage of either risk alleles thus represent an additional risk variable alongside the presence of maternally-derived non-neutralising antibodies and poor compensatory reserve.[19]–[20]

Our study has limitations. Misclassified control samples will be more common in this study than in the original GWAS of DSS cases because dengue without shock is a more common clinical outcome for a given cohort of children in an endemic area. Reassuringly, the fact that consistent associations were observed despite this limitation lends credence to our observations. In addition, as the functional basis of these mutations as yet to be clearly defined our conclusions are to an extent speculative. As dengue without shock includes a diverse range of clinical manifestations our ability to determine this functional basis is more limited.

We have shown that the *MICB* rs3132468 and *PLCE1* rs3740360 genotypes are associated with clinically apparent dengue in both adults and children, which is a significant extension from the earlier GWAS on DSS cases alone. As expected, the effect sizes of these variants is small and underscores that susceptibility to symptomatic dengue is multifactorial and includes demographic risk factors (e.g. age).[21] However, we have not performed multivariate analysis in this study as the majority of risk factors for symptomatic (non-severe) dengue are not clearly defined.. The challenge now is to define the functional basis for these observed genetic associations at *MICB* and *PLCE1* and thus increase our understanding of disease pathogenesis.

## Supporting Information

Table S1 **Details of the cohort studies used in the analysis.**
(DOCX)
