# Supplementary material for: Genetic Variants of MICB and PLCE1 and Associations with Non-Severe Dengue
Source: PLoS One. 2013 Mar 11;8(3):e59067. doi: 10.1371/journal.pone.0059067 (PMC3594159; doi:10.1371/journal.pone.0059067)
Supplement: Table S1 — Details of the cohort studies used in the analysis. (DOCX) [file pone.0059067.s001.docx]

**Supplementary Table 1: Details of the cohort studies used in the analysis**

| **Patient cohort name (sample size)** | **Inclusion criteria** | **Exclusion criteria** | **Study site and study period** | **Age Median (IQR)** | **Serotypes present** |
| --- | --- | --- | --- | --- | --- |
| BC (n=1068) | Babies born to mothers living in areas served by recruiting district hospitals | +HIV  Mothers < 15yrs | Hung Vuong Hospital, HCMC  Dong Thap Hospital, DT Province | At birth | Controls |
| FG (n=622) | Inpatients  Febrile and clinical suspicion of dengue.  Fever less than 72hrs  Age >18 months | N/A | Dong Thap Hospital, DT province  Sa Dec Hospital, DT province  Tien Giang Hospital, TG province  2007-2009 | 11 (7-14) | DENV-1 (63.7%)  DENV-2 (15%)  DENV-3 (10.8%)  DENV-4 (0.5%)  Unknown (10.1%) |
| 06DX (n=220) | Inpatients  Febrile and clinical suspicion of dengue.  Fever less than 72hrs Age < 20 years | Pregnant woman | Hospital for Tropical Diseases, HCMC  2009-2010 | 13 (11-14) | DENV-1 (60.9%)  DENV-2 (25%)  DENV-3 (10.5%)  DENV-4 (3.2%)  Unknown (0.5%) |
| 09DX (n=159) | Inpatients  Febrile and clinical suspicion of dengue.  Fever less than 72hrs  Age > 15 years | Pregnant woman | Hospital for Tropical Diseases, HCMC  2011-2012 | 23 (19-27) | DENV-1 (37.1%)  DENV-2 (45.3%)  DENV-3 (9.4%)  DENV-4 (7.5%)  Unknown (0.6%) |
| DR (n=597) | Outpatients  Febrile and clinical suspicion of dengue.  Fever < 72 hours (flexible)  Age 5 - 15 years | N/A | District 8 Hospital Outpatients, HCMC  2005-2009 | 11 (9-13) | DENV-1 (62%)  DENV-2 (15.2%)  DENV-3 (17.8%)  DENV-4 (1.2%)  Unknown (3.9%) |
| MD (n=1464) | Inpatients  Febrile and clinical suspicion of dengue.  Fever < 72 hours (flexible)  Age 5 - 15 years | N/A | Hospital for Tropical Diseases, HCMC  2001-2009 | 12 (10-13) | DENV-1 (65%)  DENV-2 (14.5%)  DENV-3 (11.7%)  DENV-4 (0.7%)  Mixed DENV (0.1%)  Unknown (8%) |
| D001 (n=76) | Clinical suspicion of dengue  < 72 hours illness: infection wards; < 6 days illness ICUs  Age 12 – 25 years | N/A | Hospital for Tropical Diseases, HCMC  2010-2011 | 18 (16-23) | DENV-1 (27.6%)  DENV-2 (30.2%)  DENV-3 (5.3%)  DENV-4 (5.3%)  DENV mixed (5.3%)  Unknown (26.3%) |
| FL (n=627) | Clinical suspicion of dengue  Any day of illness  Infection wards and ICUs  Age > 15 years | N/A | Hospital for Tropical Diseases, HCMC  2006-2008 | 21 (18-26) | DENV-1 (16.6%)  DENV-2 (10.0%)  DENV-3 (4.8%)  DENV-4 (0.3%)  Not done (68.3%) |
| DC (n=68) | Inpatients  Febrile and clinical suspicion of dengue  Any day of illness  Age <18 months | N/A | Children’s Hospital 1, HCMC  2005-2007 | 6 (4-8) months | DENV-1 (23.5%)  DENV-2 (42.6%)  DENV-3 (11.8%)  DENV-4 (1.5%)  Unknown (20.6%) |
| DT (n=93) | Inpatients  Febrile and clinical suspicion of dengue  Any day of illness  Age <18 months | N/A | Children’s Hospital 2, HCMC  2005-2007 | 7 (6-9) months | DENV-1 (34.4%)  DENV-2 (33.3%)  DENV-3 (12.9%)  Unknown (19.4%) |
| FB (n=11) | Inpatients  Febrile and clinical suspicion of dengue  Any day of illness  Age <18 months | N/A | Dong Thap Hospital, DT province  2005-2007 | 8 (7-11) months | DENV-1 (63.6%)  DENV-2 (18.2%)  Unknown (18.2%) |
| DZ (n=8) | Outpatients  Febrile and clinical suspicion of dengue  Any day of illness  Ae <18 months | N/A | Children’s Hospital 1, HCMC  2005-2007 | 8 (7-11) months | DENV-1 (12.5%)  DENV-2 (37.5%)  Unknown (50.0%) |
